# Host specialisation and disparate evolution of *Pyrenophora teres* f. *teres* on barley and barley grass

**DOI:** 10.1186/s12862-019-1446-8

**Published:** 2019-07-08

**Authors:** Celeste C. Linde, Leon M. Smith

**Affiliations:** Division of Ecology and Evolution, Research School of Biology, ANU College of Science, The Australian National University, RN Robertson Building, 46 Sullivans Creek Road, Canberra, ACT 2600 Australia

**Keywords:** Host specificity, Virulence evolution, Pathogen, Genetic diversity, Sexual reproduction

## Abstract

**Background:**

Pathogens evolve in an arms race, frequently evolving virulence that defeats resistance genes in their hosts. Infection of multiple hosts may accelerate this virulence evolution. Theory predicts that host diversity affects pathogen diversity, with more diverse hosts expected to harbour more diverse pathogens that reproduce sexually. We tested this hypothesis by comparing the microsatellite (SSR) genetic diversity of the barley leaf pathogen *Pyrenophora teres* f. *teres* (Ptt) from barley (monoculture) and barley grass (outbreeding). We also aim to investigate host specificity and attempt to track virulence on two barley cultivars, Maritime and Keel.

**Results:**

Genetic diversity in barley Ptt populations was higher than in populations from barley grass. Barley Ptt populations also had higher linkage disequilibrium levels, indicating less frequent sexual reproduction, consistent with the Red Queen hypothesis theory that genetically diverse hosts should select for higher levels of sexual reproduction of the pathogen. SSR analyses indicate that host-associated Ptt populations do not share genotypes and have independent evolutionary histories. Pathogenicity studies showed host specificity as host-associated Ptt isolates could not cross-infect hosts. Minimum spanning network analyses indicated two major clusters of barley Ptt. One cluster represents Maritime virulent and isolates from Western Australia (WA). Low *PhiPt* population differentiation between WA populations and those from Maritime and Keel, indicated a WA origin of the Maritime and Keel virulences. The main minimum spanning network cluster is represented by a panmictic population structure, represented by isolates from all over Australia.

**Conclusions:**

Although barley Ptt populations are more diverse than barley grass Ptt populations, this may be a result of the size and number of founder Ptt populations to Australia, with larger and more barley Ptt populations introduced. More frequent sexual reproduction of Ptt on barley grass support the Red Queen Hypothesis and suggest evolutionary potential of pathogens on diverse hosts are high. Extensive gene flow of Ptt between regions in Australia is suggested to maintain a panmictic population structure, with human-mediated dispersal aiding in virulence evolution of Ptt on barley.

**Electronic supplementary material:**

The online version of this article (10.1186/s12862-019-1446-8) contains supplementary material, which is available to authorized users.

## Background

The interactions between pathogens and their hosts are complex. Disease severity and incidence depend mostly on interactions between the genetic diversity and population size of hosts and pathogens, as well as on pathogen transmission ability and host composition. Disease incidence and severity can be affected by pathogen genetic diversity [1], with more genetically diverse pathogens maintaining a higher diversity of virulence. Pathogen diversity is in turn, affected by host population size, with larger host populations selecting for higher neutral diversity in the pathogen [2, 3]. Furthermore, the diversity of pathogens able to infect multiple hosts may also be higher than those able to infect a single host species only [4, 5]. Accounting for pathogen diversity and understanding the factors that drive the evolution of fungal plant pathogens are critical to identify and manage ancillary hosts for effective disease management on cultivated crops, and to better predict the emergence and spread of novel harmful genotypes [6, 7].

*Pyrenophora teres* f. *teres* (Ptt) is an important pathogen of cultivated barley, causing a disease called net form of net blotch. The disease occurs wherever barley is grown. It was first noticed on cultivated barley in western Australia in 1953 on the weedy *Hordeum leporinum* (barley grass) [8]. On barley, the sexual reproduction stage occurs on straw after harvest in autumn. Ascospores (sexual spores) are not thought to play a major role in the epidemiology of the disease, but sexual reproduction is considered important to maintain genetic diversity [9]. It is a pathogen characterized by considerable genetic diversity in Australia [10, 11] and elsewhere [12], as well as pathogenic diversity [13, 14].

Although Ptt occurs on both cultivated barley and weedy barley in Australia, host specificity for Ptt remains controversial, with some studies indicating that Ptt can infect *Hordeum* with no specificity to any particular species eg [15–17], whereas others inferred strict host specificity [18] or were inconclusive [19]. Ancillary hosts may play an important role in the evolution of pathogens on cultivated crops. A recent study on *Rhynchosporium commune,* causal agent of scald on barley, has shown that weeds harboured highly virulent strains capable of transmission to cultivated barley [3]. Therefore, weeds may threaten food security by rendering resistance genes ineffective in cultivated crops due to the evolution of new virulence. Ptt already poses a considerable threat to the barley industry because it is able to frequently defeat the resistance in barley varieties. In South Australia in 1994, Ptt re-emerged and rapidly defeated resistance in the Skiff variety, followed by the defeat of the resistance in Keel in 2007 and Maritime in 2009 [20]. It is unknown whether these virulences evolved locally or whether they were the result of pathogen migration from other barley growing regions in Australia.

Here we aim to investigate the population structure of Ptt on barley and barley grass, in all major barley growing regions of Australia using SSR markers. Barley grass (*Hordeum murinum* species complex, previously referred to as *H. leporinum* [21]) is an important and widespread weed in Australia. It is therefore, important to establish whether it is an alternate host for Ptt on barley, and thus, whether it can aid virulence evolution of Ptt. The aims for this study were to 1) Determine whether Ptt populations from barley and barley grass represent the same panmictic infective population. This will be investigated by examining allele sharing among host-associated populations using 17 microsatellite loci and with pathogenicity assays to determine cross-infectivity; 2) Characterise the genetic diversity of barley and barley grass Ptt populations in Australia using the 17 microsatellite loci; 3) Assess the diversity of host-associated Ptt populations to examine whether host diversity plays a role in pathogen diversity; 4) Determine linkage disequilibrium and mating type frequencies of Ptt host-associated populations to assess the importance of host diversity on pathogen mode of reproduction; and 5) Use this new understanding of the population structure of Ptt in Australia and tracking genotypes across Australia to shed light on the origin of Keel and Maritime virulences.

## Results

### Microsatellite diversity

In total, 298 isolates from barley grass representing nine populations and 567 isolates from cultivated barley representing 18 populations were analysed with 17 SSR loci.

### Haplotype diversity

Significantly (*P* = 0.0055) more SSR MLGs were observed in the barley Ptt population (*eMLG* = 276) than in the barley grass population (*eMLG* = 266). The Shannon-Wiener index was also higher in barley populations (*H*_*Barley*_ = 6.58, *H*_*Barley grass*_ = 5.54), with genotypes more evenly distributed in the barley grass population (*E.5*_*Barley*_ = 0.82, *E.5*_*Barley grass*_ = 0.94) (Table 1).

**Table 1:** Microsatellite diversity of *Pyrenophora teres* f. *teres* populations from barley and barley grass

								*I*_*A*_ *(P* value*)*		$\overline{r}$_*d*_ (*P* value)	
Population	N	Origin	MLG	*eMLG*	SE	*H*	*E.5*	*Not cc* ^a^	*cc* ^b^	*Not cc* ^a^	*cc* ^b^
Barley											
Keel-45	40	SA	38	9.88	0.33	3.62	0.97	0.23 (0.027)	0.19 (0.036)	0.01 (0.027)	0.01 (0.036)
Keel-55	36	SA	34	9.86	0.36	3.51	0.97	0.50 (0.001)	0.35 (0.002)	0.03 (0.001)	0.02 (0.002)
Keel-63	34	SA	29	9.43	0.71	3.28	0.86	0.92 (0.001)	0.53 (0.003)	0.06 (0.001)	0.03 (0.003)
Keel-64	28	SA	27	9.88	0.32	3.28	0.98	1.18 (0.001)	1.19 (0.001)	0.08 (0.001)	0.08 (0.001)
Maritime-46	40	SA	30	9.14	0.84	3.27	0.82	0.89 (0.001)	0.47 (0.001)	0.06 (0.001)	0.03 (0.001)
Maritime-47	38	SA	32	9.57	0.61	3.40	0.92	0.41 (0.002)	0.22 (0.040)	0.03 (0.002)	0.01 (0.040)
Maritime-48	40	SA	31	9.07	0.90	3.27	0.75	0.47 (0.001)	0.25 (0.035)	0.03 (0.001)	0.02 (0.035)
Maritime-56	40	SA	35	9.67	0.55	3.50	0.92	0.66 (0.001)	0.48 (0.001)	0.04 (0.001)	0.03 (0.001)
Maritime-57	39	SA	39	10.00	0.00	3.66	1.00	0.61 (0.001)	0.61 (0.001)	0.04 (0.001)	0.04 (0.001)
Maritime-58	37	SA	37	10.00	0.00	3.61	1.00	0.39 (0.001)	0.39 (0.002)	0.02 (0.002)	0.02 (0.002)
NSW	21	NSW	21	10.00	0.00	3.04	1.00	1.32 (0.001)	1.32 (0.001)	0.08 (0.001)	0.08 (0.001)
Old_SA	24	SA	22	9.67	0.51	3.06	0.96	1.39 (0.001)	1.23 (0.001)	0.09 (0.001)	0.08 (0.001)
Qld	31	Qld	30	9.90	0.30	3.39	0.98	1.09 (0.001)	0.86 (0.001)	0.07 (0.001)	0.05 (0.001)
SA_GP	14	SA	12	9.01	0.66	2.44	0.94	2.31 (0.001)	1.85 (0.001)	0.15 (0.001)	0.12 (0.001)
SA_PV_B	35	SA	35	10.00	0.00	3.56	1.00	0.93 (0.001)	0.89 (0.001)	0.06 (0.001)	0.06 (0.001)
SA09	13	SA	13	10.00	0.00	2.56	1.00	2.82 (0.001)	2.82 (0.001)	0.18 (0.001)	0.18 (0.001)
Vic	11	Vic	11	10.00	0.00	2.40	1.00	0.76 (0.001)	0.76 (0.001)	0.05 (0.001)	0.05 (0.001)
WA	46	WA	46	10.00	0.00	3.83	1.00	0.11 (0.464)	0.11 (0.464)	0.01 (0.464)	0.01 (0.464)
***Barley Total***	***567***		***506***	***276.24***	***3.59***	***6.15***	***0.82***	***1.00*** ***(0.001)***	***0.88 (0.001)***	***0.06 (0.001)***	***0.06 (0.001)***
BG_Barm	28	NSW	27	9.88	0.32	3.28	0.98	−0.04 (0.643)	−0.08 (0.806)	0.00 (0.643)	−0.01 (0.806)
BG_Fin	27	NSW	26	9.87	0.33	3.24	0.98	0.02 (0.39)	−0.02 (0.564)	0.00 (0.39)	0.00 (0.564)
BG_Kat	42	WA	36	9.69	0.52	3.54	0.95	0.05 (0.438)	0.04 (0.545)	0.01 (0.438)	0.01 (0.545)
BG_Nar	57	NSW	42	9.48	0.67	3.64	0.89	0.36 (0.001)	0.16 (0.210)	0.04 (0.001)	0.16 (0.210)
BG_SA_DOW	49	SA	49	10.00	0.00	3.89	1.00	0.61 (0.001)	0.61 (0.001)	0.04 (0.001)	0.04 (0.001)
BG_SA_PV	17	SA	16	9.67	0.47	2.75	0.97	2.25 (0.001)	2.15 (0.001)	0.16 (0.001)	0.16 (0.001)
BG_Tem	10	NSW	8	8.00	0.00	1.97	0.85	0.78 (0.003)	−0.15 (0.704)	0.08 (0.003)	−0.02 (0.704)
BG_WW_GR	38	NSW	34	9.74	0.47	3.49	0.95	0.04 (0.519)	0.05 (0.516)	0.00 (0.519)	0.00 (0.516)
BG_WW_MR	30	NSW	29	9.90	0.30	3.35	0.98	0.08 (0.310)	0.06 (0.345)	0.01 (0.310)	0.01 (0.345)
***Barley grass Total***	***298***		***266***	***266.00***	***0.00***	***5.54***	***0.94***	***0.58 (0.001)***	***0.54 (0.001)***	***0.04 (0.001)***	***0.04 (0.001)***

*SA* South Australia, *NSW* New South Wales, *Vic* Victoria, *WA* Western Australia, *Qld* Queensland

N = Number of Ptt isolates analysed

*eMLG* = The number of expected MLGs at the smallest sample size based on rarefaction [22] with standard error (SE)

*H* = Shannon-Wiener Index of MLG diversity [23]

*E.5* = Evenness, ie equitability in the distribution of the sampling units [23, 24]

Linkage disequilibrium indices $\overline{r}$_*d*_ [25] and the index of association (*I*_*A*_) [26]

^a^Not cc = non-clone corrected data set

^b^cc = clone corrected data set

**Fig. 1:**
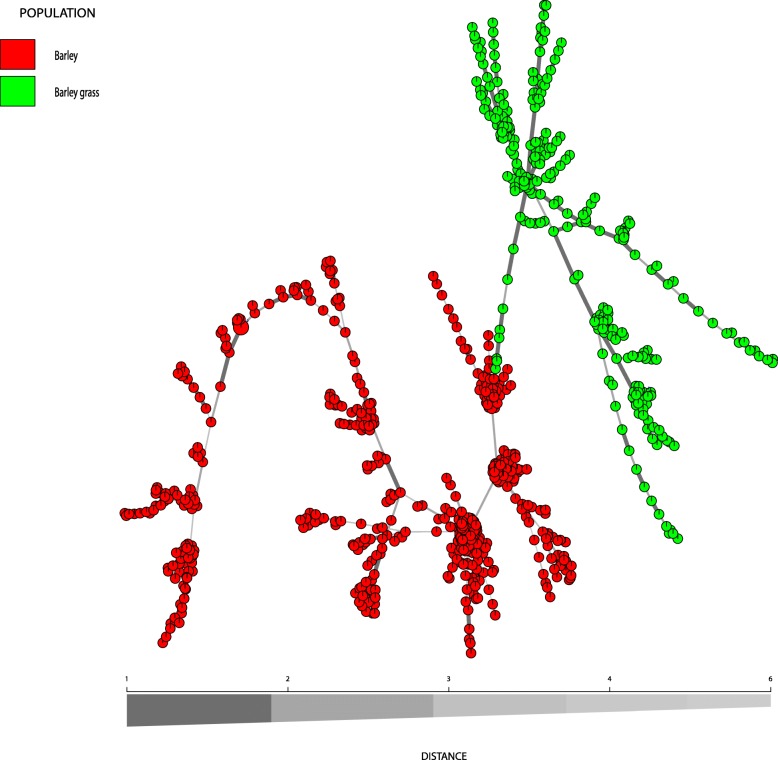
Minimum spanning network based on a dissimilarity matrix using Bruvo’s distance as calculated in Poppr. Only the 772 MLGs observed in Ptt populations from barley and barley grass in Australia are displayed. Node colours represent population membership. Edge (line) thickness and shading represent relatedness between MLGs. Edge length is arbitrary

**Fig. 2:**
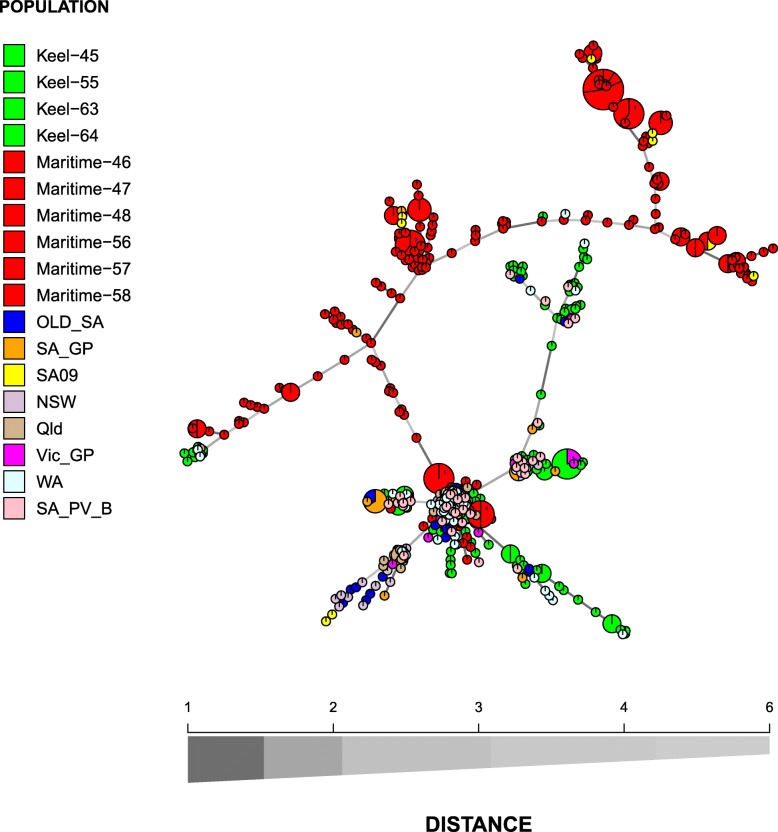
Minimum spanning network based on a dissimilarity matrix using Bruvo’s distance as calculated in Poppr. All MLGs of the 567 analysed Ptt isolates representing populations from barley are displayed. Node colours represent population membership. All populations from Keel and Maritime are displayed in green or red to assist in visual comparison of these populations with the rest. Edge (line) thickness and shading represent relatedness between MLGs. Edge length is arbitrary

**Fig. 3:**
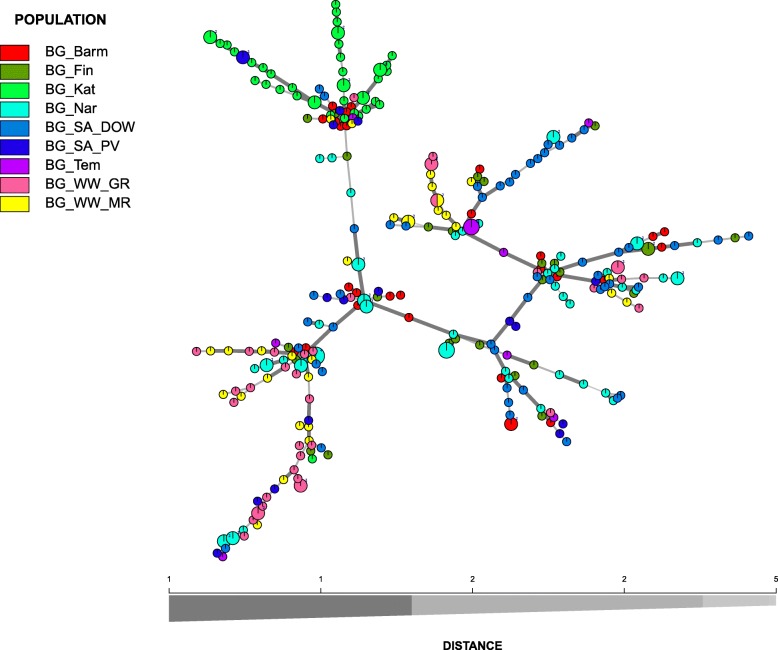
Minimum spanning network based on a dissimilarity matrix using Bruvo’s distance as calculated in Poppr. All MLGs of the 298 analysed Ptt isolates representing populations from barley grass are displayed. Node colours represent population membership. Edge (line) thickness and shading represent relatedness between MLGs. Edge length is arbitrary

No MLGs were shared between host-associated populations (Fig. 1). Nine MLGs were shared among barley populations (all populations were within South Australia; shared MLGs between Maritime or Keel populations are not shown). Maritime and Keel populations did not share MLGs (Fig. 2). One MLG was shared among barley grass populations (two populations from Wagga Wagga, NSW) (Fig. 3).

### Allelic diversity

Gene diversity in Ptt populations from barley was high and ranged from *H*_*e*_ = 0.42 to 0.64, and *H*_*e*_ = 0.66 for all populations from barley combined. In contrast, *H*_*e*_ for populations from barley grass ranged from 0.20 to 0.44, and *H*_*e*_ = 0.40 for all barley grass populations combined. Most individual populations as well as the combined population from barley had significantly higher gene diversity (*H*_*e*_) (*P* = 0.0001), more alleles (*P* = 0.0466), more effective alleles (*P* = 0.0060) and higher Shannon information indices (*P* = 0.0008) than populations from barley grass (Table 2). *P*-values pertain to pairwise comparisons of indices for total host-associated populations. More private alleles were observed in the barley (115) compared to barley grass (26) populations.

**Table 2:** Microsatellite diversity (17 loci) for *Pyrenophora teres* f. *teres* populations from barley and barley grass

Population	Na	SE	*Ne*	SE	*I*	SE	*H*_*e*_	SE
Barley								
Keel-45	4.35	0.45	2.79	0.35	1.05	0.11	0.57	0.04
Keel-55	4.24	0.52	2.59	0.28	1.00	0.11	0.54	0.05
Keel-63	3.94	0.40	2.39	0.27	0.95	0.10	0.51	0.05
Keel-64	3.59	0.45	2.39	0.23	0.92	0.11	0.51	0.05
Maritime-46	3.53	0.59	2.01	0.17	0.79	0.10	0.45	0.04
Maritime-47	3.18	0.31	1.87	0.14	0.73	0.06	0.43	0.04
Maritime-48	3.41	0.42	1.93	0.18	0.74	0.08	0.42	0.04
Maritime-56	4.35	0.54	2.09	0.14	0.90	0.08	0.48	0.04
Maritime-57	3.65	0.44	1.96	0.13	0.82	0.07	0.46	0.04
Maritime-58	3.53	0.39	2.13	0.17	0.86	0.08	0.48	0.04
SA_Old	3.47	0.26	2.43	0.17	0.97	0.07	0.56	0.03
SA_GP	4.06	0.37	2.82	0.31	1.09	0.10	0.58	0.04
SA09	4.12	0.37	3.14	0.27	1.19	0.09	0.64	0.03
NSW	4.06	0.36	2.58	0.21	1.04	0.09	0.56	0.04
Qld	4.82	0.39	3.04	0.24	1.22	0.08	0.64	0.03
Vic_GP	3.00	0.19	2.14	0.13	0.85	0.07	0.50	0.04
WA	4.94	0.76	2.72	0.32	1.08	0.11	0.57	0.04
SA_PV_B	4.77	0.60	2.81	0.38	1.06	0.13	0.55	0.05
***Barley total***	***9.59***	***1.40***	***3.27***	***0.31***	***1.33***	***0.10***	***0.67***	***0.03***
Barley grass								
BG_Barm	2.53	0.32	1.87	0.21	0.61	0.12	0.35	0.07
BG_Fin	2.47	0.37	1.75	0.18	0.55	0.12	0.33	0.07
BG_Kat	2.24	0.58	1.57	0.30	0.37	0.13	0.20	0.06
BG_Nar	2.53	0.34	1.77	0.18	0.57	0.12	0.33	0.07
BG_Tem	2.00	0.26	1.62	0.14	0.49	0.10	0.31	0.06
BG_WW_MR	2.47	0.33	1.71	0.18	0.54	0.11	0.31	0.07
BG_WW_GR	2.59	0.29	1.68	0.15	0.55	0.10	0.33	0.06
BG_SA_PV	3.06	0.36	2.18	0.27	0.79	0.12	0.44	0.06
BG_SA_DOW	3.29	0.29	1.68	0.15	0.58	0.10	0.32	0.06
***Barley grass total***	***5.47***	***0.99***	***2.01***	***0.22***	***0.75***	***0.13***	***0.40***	***0.07***
**Mean**	**3.49**	**0.09**	**2.21**	**0.05**	**0.83**	**0.02**	**0.46**	**0.01**

*Na* number of alleles, *Ne* effective number of alleles, *I* Shannon’s information index [27], *H*_*e*_ Nei’s gene diversity [28]. Each index is followed by *SE* standard error, in the succeeding column

### Population structure

Population differentiation (*PhiPt*) in pairwise comparisons were all significant (*P* < 0.05 or 0.01), except for a number of pairwise comparisons among barley grass Ptt populations from NSW. Those include pairwise comparisons of BG_Tem with BG_Fin, BG_Barm and BG_Nar. Some pairwise comparisons between populations from Maritime (Maritime_58 and Maritime_57, Maritime_47 and Maritime_48) also did not show significant *PhiPt* values (Additional file 1: Table S2).

Populations from Keel showed lowest *PhiPt* values in pairwise comparisons with WA (*PhiPt* = 0.039–0.068). Maritime populations showed lowest *PhiPt* values with SA09 (*PhiPt* = 0.087–0.132), followed by WA (*PhiPt* = 0.204–0.252) (Additional file 1: Table S2). In the AMOVA, most genetic diversity (55%) was attributable to differences among individuals within populations (*PhiPT* = 0.452), with a considerable share of the total diversity (31%) attributable to differences between the two hosts-associated populations (*PhiRT* = 0.315) (Table 3). Accordingly, pairwise population differentiation between host-associated populations was high (*PhiPt* = 0.306, *P* = 0.001). Differentiation among barley Ptt populations accounts for only 22% (*PhiPT* = 0.218, *P* = 0.001) of the variation among all populations, whereas variation among barley grass populations accounts for 19% (*PhiPT* = 0.194, *P* = 0.001) (Additional file 1: Table S3).

**Table 3:** Hierarchical Analyses of Molecular Variance partitioning of *Pyrenophora teres* f. *teres* SSR data among and within hosts and populations

Source	df	SS	MS	Estimated variance	Percentage variance	AMOVA statistics	*P*
Between host groups	1	989.367	989.367	2.420	31%	*PhiRT* = 0.315	0.001
Within hosts	25	940.771	37.631	1.056	14%	*PhiPR* = 0.200	0.001
Within populations	838	3529.534	4.212	4.212	55%	*PhiPT* = 0.452	0.001

*P*-value estimates are based on 999 permutations. *df* degrees of freedom, *SS* sum of squares, *MS* mean squared deviations

**Fig. 4:**
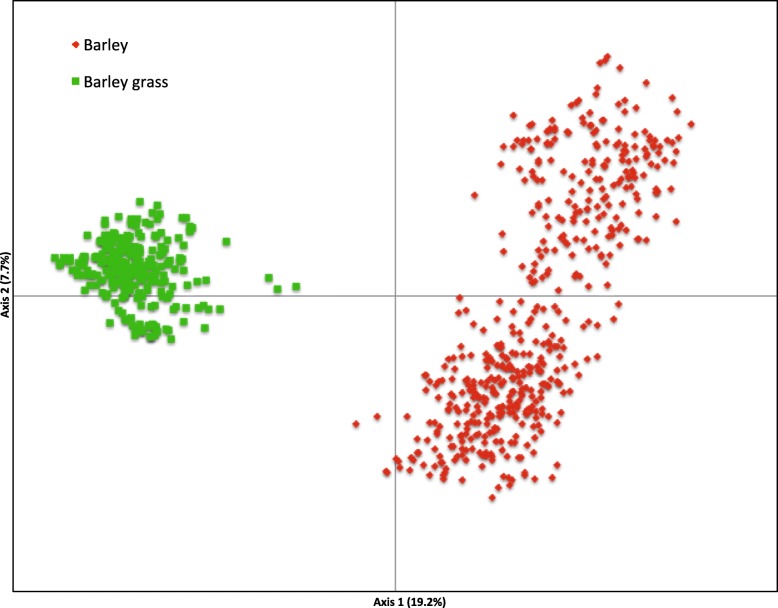
Scatter plot of the first two components of the principle coordinates analyses of the 772 multilocus genotypes of *Pyrenophora teres* f. *teres* isolates from barley and barley grass in Australia

Principle component analyses (PCoA) clearly separated the barley and barley grass MLGs into separate clusters, with axis 1 and 2 accounting for 19.2 and 7.7% respectively of the genetic variability (Fig. 4), indicating a moderate genetic structure.

### Linkage disequilibrium

Only the populations that were collected in transects from a barley paddock or barley grass field, and not merely as a number of isolates representing a region, should be considered for linkage disequilibrium analyses due to a possible Wahlund effect. Thus eligible populations include those from Maritime, Keel and the SA_PV_B population from barley, as well as all the populations from barley grass. All eligible clone corrected and non-clone corrected barley Ptt populations were in significant linkage disequilibrium as measured with the *I*_*A*_ and $\overline{r}$_*d*_ statistics. In contrast, only two (BG_SA_DOW and BG_SA_PV) of the nine barley grass Ptt populations were in significant linkage disequilibrium (*P* = 0.001) (Table 1).

### Mating type frequencies

In all 10 analysed barley Ptt populations, mating type frequencies did not deviate from a 1:1 ratio. Similarly, barley grass Ptt mating type frequencies, except for population BG_WW_MR, did not deviate significantly from a 1:1 ratio (Table 4).

**Table 4:** Mating type frequencies of *Pyrenophora teres* f. *teres* in populations from barley and barley grass in Australia

Population	N Mat1–1	N Mat1–2	N (sample size)	Chi square	Significance
Barley					
Keel-45	15	14	29	0.035	ns
Keel-55	19	12	31	1.581	ns
Keel-63	19	9	28	3.571	ns
Maritime46	23	14	37	2.189	ns
Maritime-47	15	17	32	0.125	ns
Maritime-48	15	15	30	0.000	ns
Maritime-56	15	20	35	0.714	ns
Maritime-57	18	19	37	0.027	ns
Maritime-58	6	9	15	0.600	ns
SA_PV_B	19	16	37	0.257	ns
Barley grass					
BG_Nar	15	23	38	1.684	ns
BG_Kat	10	12	22	0.182	ns
BG_Fin	13	13	26	0.000	ns
BG_Barm	16	11	27	0.926	ns
BG_WW_MR	23	6	29	9.966	*P* < 0.01
BG_WW_GR	16	18	34	0.118	ns
BG_SA_Dow	18	17	35	0.029	ns
BG_SA_PV	9	7	16	0.250	ns

### Evolutionary relationship among MLGs

No shared MLGs are observed among host populations and host populations clustered separately (Fig. 1). Evolutionary relationships among MLGs as visualized with a minimum spanning network revealed some geographic structuring for Ptt isolates from barley and barley grass, most notably for WA isolates from barley grass (BG_Kat). However, there were exceptions for all populations (Figs. 2-3).

Some genetic structuring is visible in barley Ptt with most isolates from the cultivar Maritime clustering separately from the rest. Only eight Keel MLGs clustered together with Maritime isolates, as well as three MLGs from WA, seven from SA09, two from Queensland and 1 MLG from SA_GP (Fig. 2). Most of the isolates that clustered together with Maritime isolates, were sampled in the same year, ie 2009, or thereafter (SA_GP, Qld) (Fig. 2).

### Pathogenicity

Barley Ptt isolates were all pathogenic to at least two of the six barley cultivars. Similarly, barley grass Ptt were pathogenic to at least one barley grass line. Infection scores of cross-host infections ranged from 0 to 2 (Additional file 1: Table S4), indicative of strict host specificity.

## Discussion

In this study, we examined 865 Ptt isolates from barley and barley grass, representing a large number of populations in Australia using 17 unlinked [29] SSR loci. Following on from our previous studies on *Rhynchosporium commune* [3], we investigated the hypothesis that host genetic diversity and pathogen population size affect pathogen diversity. Furthermore, we investigated the controversial issue of host specificity in Ptt, and attempted to infer the origin of two virulence types in South Australia. This study represents the largest and most comprehensive population study of Ptt to date and significantly improves our understanding of its population genetic biology.

### Does the host make-up affect pathogen genetic diversity and mode of reproduction?

Numerous studies have investigated the population genetic structure of Ptt in Australia and elsewhere eg. [10–12]. While the genetic markers differed in most of these studies, and the findings are not directly comparable, Ptt population genetic diversity has always been reported as high regardless of genetic marker used. However, none of the previous studies have investigated Ptt populations from barley grass to compare the effect of host on pathogen diversity.

A recent study [3] on another leaf pathogen of barley, *Rhynchosporium commune*, suggested that pathogen population size, rather than host diversity, is a better predictor of the patterns of pathogen neutral genetic diversity. Similarly, *Zymoseptoria tritici* shows evidence for faster genomic evolution and a higher effective population size on cultivated wheat compared to its sister species on wild grasses [30]. In the present study of Ptt from barley, all neutral genetic diversity estimates based on the 17 SSR loci, including the number of *eMLG*s and estimates of number of alleles, effective number of alleles, Nei’s gene diversity and the Shannon’s information index, were significantly higher than for populations from barley grass. This further suggest that the genetic diversity of the pathogen is not affected by host diversity.

Small pathogen population size has been suggested to explain low pathogen diversity in weeds [3]. In the case of *R. commune* on barley, the incidence of the pathogen is low on barley grass, hence pathogen population size (N) is low on the weedy host. Similarly, *Zymoseptoria* population size (N) on wild grasses would be lower than on cultivated wheat. In contrast, Ptt is present in almost all populations of barley grass investigated (data not shown). Coupled with barley grass being a prolific weed in barley growing areas of Australia, N of Ptt on barley grass is considered as comparable or even higher than on barley. On barley, Ptt is often chemically controlled or resistant barley cultivars are used, reducing N of barley-Ptt in Australia. This study, therefore, does not support the findings of Linde et al. [3] that pathogens with a large population size have higher levels of genetic diversity.

It is perhaps not surprising that we do not find consistent patterns between host diversity, pathogen N and pathogen diversity, as changes in genetic diversity are likely to occur over a long evolutionary time frame. Cultivated crop pathogens are almost always founder populations that do not have a long evolutionary history with their host in the region studied. Instead, the genetic signature we typically observe is largely a function of the number and size of founder populations. Pathogens associated with cultivated crops usually have a higher probability of being introduced as founder populations due to seed importation associated with eg. breeding programs [31], than pathogens of weeds. In contrast, it is unlikely that numerous pathogen populations of barley grass were introduced to Australia, as it is not a commercially important crop. Thus, the higher diversity of the barley pathogen population is likely because of the establishment of larger founder populations.

Most fungi reproduce both asexually and sexually, with fungi that have a mixed reproduction system considered to pose a higher evolutionary risk, and predicted to overcome newly deployed resistance genes more rapidly than those that can reproduce only either asexually (clonal) or sexually [1]. This is because during sexual reproduction, virulence alleles from two individuals can be combined into the same genetic background and asexual reproduction rapidly propagates such new gene combinations, while keeping well-adapted gene combinations together. By extension of the Red Queen Hypothesis [32, 33], it is predicted that pathogens which infect sexual hosts should be genetically more diverse than those infecting asexual hosts. Thus, it is expected that sexual reproduction in pathogens should be more common on the more diverse host (eg. in barley grass). Also, sex in fungi is often triggered by stress [34], which is predicted to be higher in non-cultivated host populations (eg. barley grass). On barley grass, most populations show equal mating type frequencies (except BG_WW_GR) and only two of the nine populations are in linkage disequilibrium (non-random mating). In contrast, all 11 of the eligible populations from barley show significant linkage disequilibrium (Table 1), although equal mating type frequencies were observed (Table 4). This expected finding of linkage disequilibrium of the pathogen on the less diverse host was not shown for *R. commune* [3] where there was a likely interplay between host diversity and pathogen population size. This study therefore, supports the Red Queen Hypothesis [32, 33], by providing evidence for genetically diverse (outcrossing) hosts maintaining sexual reproduction in their pathogens.

It is also expected that populations that reproduce sexually have higher genotypic diversity [35]. Here, despite evidence for random mating ie linkage equilibrium among SSR loci, genotypic diversity in barley grass Ptt populations are lower than in barley Ptt populations, based on genotypic diversity (*eMLG* and Shannon-Wiener diversity, *H*), but also on gene diversity (*He*, *I*, number of alleles) estimates. Again, this is likely a function of the size and number of founder populations introduced to Australia, where we predict that barley Ptt founder populations were larger and were introduced more often than barley grass Ptt populations.

### Host specificity and evolutionary history of Ptt populations from barley and barley grass

Host specialisation, coupled with geographic separation, form gene flow barriers that will lead to host differentiated pathogen populations [36]. When geographic separation is absent, such as in Ptt on barley and barley grass, gene flow will allow these lineages to combine. Hybridisation between such lineages may lead to new disease properties such as increased virulence or new host affinities [6, 37–39]. To limit virulence evolution, it is thus important to identify whether Ptt host-associated lineages can co-occur on the same host.

Host specificity in Ptt has a long and controversial history. Non-*Hordeum* species such as *Bromus diandrus* in Western Australia [40] and *Avena sativa* (oats) in Finland [41] were reported as susceptible to *Pyrenophora teres*. Kenneth [17] even reported that isolates from wild barley (eg *Hordeum leporinum*) were able to infect cultivated barley and vice versa. Unfortunately, these latter studies did not specify whether they used Ptt or Ptm (*Pyrenophora teres* f. *maculata*). Subsequently, a study in Australia [18] reported strict host specificity with Ptt isolates from barley and *H. leporinum* unable to cross-infect. Khan [18] used host specific isolate mixtures in his pathogenicity trials with 15 isolates from barley and seven from barley grass tested. Brown et al. [19] could not replicate infection of *Avena sativa* and most species of *Bromus* with Ptt isolates from barley, but expanded the host range for Ptt isolates from barley to 85 host species, including species of *Hordeum* (including *H. murinum*), *Agropyron*, *Elymus*, *Hordelymus* and *Stipa*. They did report “reduced virulence” of a barley grass isolate on barley and were inconclusive whether *H. murinum* subsp. *leporinum* (barley grass) is indeed a susceptible host for Ptt isolates from barley. Recently, another Australian study [13] reported an isolate HRS#10128 from barley grass virulent on barley cultivars representing 3 of the 7 tested barley line groups tested (groups of barley cultivars responded similarly to Ptt infection).

In this study, pathogenicity assays clearly showed host specificity in Ptt, ie none of the barley grass lines were susceptible to Ptt isolates from barley, and none of the barley cultivars were susceptible to barley grass isolates, although all isolates were pathogenic on some of the cultivars/lines of the host they were originally isolated from. Previous studies on host specificity in this system either did not distinguish between Ptt and Ptm [17], or used a single isolate from barley grass only [13] which may have been misidentified. This study used 10 isolates from each host, confirmed as Ptt with a species-specific PCR test, and therefore provides unequivocal evidence for host specificity. Pathogenicity studies using 20 additional Ptt isolates from each host confirmed this finding (data not shown).

The two host-associated populations of Ptt were clearly separated into two distinct clusters with a PCoA (Fig. 4). Also, there was high and significant population genetic differentiation (*PhiPt* = 0.306, *P* = 0.001) between them, and the AMOVA showed that much of the total genetic diversity (31%, *PhiRT* = 0.315) was attributable to differences between the host-associated populations. A minimum spanning network further showed that isolates from the two hosts have a different evolutionary history, despite being sampled from similar geographic areas (Figs. 1-3). Differentiation based on host species was previously shown using AFLPs, but for only a small number of isolates [42]. In this study, we genotyped 865 isolates and found strong evidence for a host-associated pattern in population genetic structure. Undoubtedly, the host specificity prevents genetic exchange between host-associated Ptt populations, despite occurring in sympatry as was also found for *Botrytis cinerea* [43]. Novel resistance genes are commonly found in wild *Hordeum* accessions [44, 45] and thus host specificity is likely achieved by the difference in host resistance genes. Also, toxin composition [46–48] of host-associated Ptt populations may differ, which may contribute to host specificity [49].

### Can we elucidate the origin of maritime and keel virulent strains?

Tracing the origin of virulence in plant pathogens is valuable to identify routes of pathogen transfer. However, this is far easier achieved in pathogens that reproduce as clonal lineages, such as *Phytophthora ramorum* [50, 51], than in genetically diverse pathogens with high rates of recombination, such as in Ptt. Genetic relatedness as visualised by the minimum spanning network analyses, suggested the Maritime virulent isolates (on a distinct branch) had a separate evolutionary history to most of the isolates analysed in this study. A number of isolates from SA09 collected in South Australia in the same year the Maritime virulence became epidemic (2009), clustered together with the Maritime isolates (Fig. 2). Since the majority of Ptt isolates, excluding most of the Maritime isolates, formed one large panmictic population, these SA09 isolates most likely already represent an introgression with the Maritime isolates. Similarly, the Maritime isolates, which clustered with the rest of the Ptt isolates, have already introgressed into the panmictic population. However, some isolates from WA also clustered with the Maritime virulent populations (Fig. 2). Also, population differentiation between WA and Maritime populations are the lowest (*PhiPt* = 0.204–0.252), discounting the SA09 population as discussed above. South Australian Ptt isolates sampled prior to 2009, indeed showed much higher *PhiPt* values with Maritime populations, suggesting the virulence did not evolve locally in South Australia. Together, network analyses and population genetic differentiation suggest that the Maritime virulence evolved in WA and was then introduced to South Australia.

The evolutionary patterns for the Keel virulence isolates were less clear in the network analyses, although the lowest *PhiPt* values in pairwise population comparisons were achieved between Keel and WA isolates (*PhiPt* = 0.039–0.068). This suggests that the Keel virulence was also introduced from WA to South Australia. With the exception of the Maritime and some WA isolates discussed above, the majority of isolates analysed, from different parts of Australia, formed one large panmictic population (Fig. 2). This is indicative of a pathogen with high levels of gene flow, achieved by a combination of natural and human-mediated dispersal.

Despite inferred high levels of gene flow in Ptt, the mechanism of the Maritime and Keel virulence introduction from WA to South Australia is unclear. Although the Maritime epidemic had a eastward trajectory as it moved rapidly from the far west coast of South Australia across the Eyre Peninsula and onto Yorke Peninsula [20], WA and South Australia is divided by the Nullarbor Plain, which is an arid region void of barley production. It is thus unlikely that natural dispersal of the pathogen resulted in the migration event, as dispersal distance is undoubtedly less than 500 km [1], a conservative estimate of the distance between the closest barley fields in WA and South Australia. The only possible mechanism for this dispersal is human-mediated gene flow, perhaps in the form of hay or infected seed [52].

## Conclusions

In summary, surprisingly, Ptt populations are more genetically diverse on the less genetically diverse host which likely harbours a smaller pathogen census population size [3], but as expected show more sexual reproduction on the genetically diverse host [32, 33]. Furthermore, strict host specificity and separate evolutionary histories suggest that the Ptt host-associated populations should be treated as non-interacting entities. The contrasting patterns of evolution suggest that different founder histories affected those inferences, where Ptt on barley were likely introduced more often and from multiple source populations. We also suggest that the Maritime and Keel virulence evolved in Western Australia, after which human-mediated migration introduced the virulent strains to South Australia. The erosion or defeat of Ptt resistance in barley is a far too common occurrence [20, 53, 54]. In Australia, this led to a race between barley breeders and the evolution of the pathogen, indicating the barley industry is vulnerable to net form of net blotch [20]. Although we have shown that barley grass is not a host for Ptt from barley, other ancillary hosts may select for high pathogen race diversity [55, 56]. Resistance genes in barley grass should be explored to combat the disease in barley. As such, understanding the evolution of the pathogen on barley and other hosts, and strategies aimed at resistance breeding are increasingly important.

## Methods

### Host diversity and pathogen census population size

Cultivated barley (*Hordeum vulgare*) is considered to be genetically less diverse than barley grass. Genetic diversity in *Hordeum murinum*, unlike the monocultures of cultivated barley, is high because of regular outcrossing (see refs within [3] suggesting outcrossing) and little erosion of genetic diversity due to human selection and domestication. Population size (N) of the pathogen is difficult to estimate. Unlike *R. commune* [3], Ptt is commonly found in Australia on barley grass in most populations analysed. In contrast, Ptt on barley is often controlled by either chemical applications or use of resistant cultivars. Because barley grass is a common weed in most areas where barley is grown, we therefore assume that N of barley grass Ptt populations are equivalent or even higher than for Ptt on barley.

### Microsatellite analyses

Seventeen of the 20 previously developed neutral microsatellite or simple-sequence-repeat loci (SSR) [29], excluding loci Pt129631, Pt144681 and Pt243550 were used to characterize a total of 298 Ptt isolates from barley grass and 567 from barley. Eighteen populations from barley and nine from barley grass were analysed (Table 1, Additional file 1: Table S1). Of the barley populations, four were from the barley cultivar Keel and six from Maritime. These South Australian populations were collected in 2009, thus only 2 years after the virulence appeared on Keel, and the same year the epidemic occurred on Maritime in South Australia. Diseased leaves for each population were collected from a 1 × 50 m transect for isolation of *Pyrenophora* from barley and barley grass. One isolate per infected leaf was collected 1 m apart to give 10 to 59 Ptt isolates collected per transect. For *Pyrenophora* populations from barley, only the Keel, Maritime and the SA_PV_B populations from barley were sampled following a transect, with all the other barley Ptt populations obtained from collaborators, thus representing a large geographic area and diverse timeline, except for SA09 which was collected in 2009 from South Australia, but does not represent a transect sampling. SSR loci and fragment analyses followed [29] for Ptt SSR loci.

All isolates were confirmed as Ptt using the PTT-F & PTT-R as well as PTM-F & PTM-R primers from Williams et al. [57]. PTM/PTT PCRs were performed in 30 μl reactions using 1x MangoTaq buffer (Bioline), 2.2 mM MgCl_2_, 125 mM dNTPs,0.5 μM each primer, 0.5 mg/mL BSA, ~ 10 ng genomic DNA and 0.03 U/μL MangoTaq (Bioline, London, UK) with touchdown thermal cycling: 3 min at 95 °C, followed by 10 cycles of 94 °C for 30 s, 65 °C (− 1 °C/cycle) for 45 s and 72 °C for 30 s and a further 25 cycles of 94 °C for 10 s, 56 °C for 15 s, 72 °C for 30 s).

SSR locus PCRs were performed in 30 μL reactions using iTaq (Scientifix, Cheltenham, Australia) buffer at 1x concentration with 2 mM MgCl_2_, 125 mM dNTPs, 0.125 μM untailed locus-specific primer, 0.012 μM M-13 tailed locus-specific primer, 0.25 μM fluorescently labelled M13 primer, 0.5 mg/mL BSA, ~ 10 ng genomic DNA and 0.02 U/μL Mango Taq (Bioline, London, UK). PCR cycling conditions were 3 min at 95 °C, followed by 35 cycles of 30 s at 94 °C, 30 s at 58 °C and 30 s at 72 °C. Concentration of PCR products were estimated on 1.6% agarose gels and equivalent concentrations of each product were pooled into 10 μL volume and cleaned up using 4uL AmpureXP magnetic beads (Beckman Coulter Inc., Brea, USA) with 14uL binding buffer (20% PEG8000, 2.5 M NaCl), washed twice with 150 mL 70% ethanol and eluted in 25 mL TE. Six microliters of this SSR solution was mixed with 9 mL HiDi (Thermo Fisher, Waltham USA) and 3 μL 1:25 diluted (in HiDi) 600LIZ size standard (Thermo Fisher). This mixture was denatured for 3 min at 95 °C and run on an ABI 3130XL Genetic Analyzer. DNA extraction and SSR analyses for a subset of 20 isolates from each host were repeated to check reproducibility of all SSR loci.

### Microsatellite diversity

#### Haplotype diversity

We used the methodology previously described by Linde et al. [3] to define multilocus genotypes (MLGs) and calculate a number of indices describing MLG occurrences and distributions, using the R package *Poppr* [58]. Indices calculated include the number of MLGs in each individual and host-associated population and the expected number of MLGs after rarefaction (*eMLG*), the Shannon-Wiener index (*H*) of MLG diversity [23, 24] and the evenness index *E.5* [23, 24]).

#### Allelic diversity

GenAlEx v6.502 [59, 60] was used to calculate estimates of neutral genetic diversity at the population and host-associated population level. Estimates included the number of alleles (*N*_*a*_), number of private alleles, Nei’s gene diversity (*H*_*e*_) [28] and Shannon’s information index (*I*) [27]. T-tests were performed to compare the means of these statistics in pairwise comparisons of host-associated populations.

#### Population structure

To investigate whether Ptt from barley and barley grass belong to divergent genetic pools, population structure was assessed with an AMOVA and principle coordinates analysis (PCoA) using GenAlEx v6.502 [59, 60]. Only one individual from each MLG was used in the PCoA analyses. Significance for the AMOVA and population differentiation (*PhiPt*) between pairwise comparisons of population was determined by 999 permutations.

#### Linkage disequilibrium

Analyses for linkage disequilibrium to test for a random association among SSR alleles of host-associated Ptt populations were applied by calculating the index of association (*I*_*A*_) [26] and the standardized index of association $\overline{r}$_*d*_ [25] in the R package *Poppr* [58], following Linde et al. [3].

#### Evolutionary relationship among MLGs

To assess the evolutionary relationships among Ptt MLGs, minimum spanning networks were constructed using the function *poppr.msn* [58] in R. The network was constructed based on Bruvo’s distance [61], a simple method for the calculation of microsatellite genotype distances irrespective of ploidy level. Data were grouped to represent individuals and populations from barley, as well as populations representing the two host groups. Networks were constructed using clone corrected SSR data. Non-clone corrected SSR data was used to construct networks for barley and barley grass Ptt populations separately. The networks were visualized using the package *igraph* [62].

#### Mating type frequencies

MAT1–1 and MAT1–2 loci were amplified for a selected number of Ptt populations from barley and barley grass. MAT1–1/MAT1–2 PCRs were performed using primers PtMAT-F & -R from Lu et al. [63]. PCRs were performed in 30 μL reactions using 1x MyTaq buffer (Bioline), 0.35 μM each primer, ~ 10 ng genomic DNA and 0.025 U/uL MyTaq (Bioline). Thermal cycling conditions were 3 min at 95 °C, followed by 35 cycles of 94 °C for 20 s, 55 °C for 30 s and 72 °C for 30 s. Mating type frequency analyses were performed on clone corrected population data sets only. Proportions of MAT1–1 and MAT1–2 isolates were calculated for each population studied. A χ^2^ test [64] was used to determine whether departures from a 1:1 frequency were significant, indicating departures from sexual reproduction.

### Pathogenicity

#### Isolates and seed types

Ten isolates of *Pyrenophora* from each of *H. leporinum* (barley grass) and *H. vulgare* (barley), collected between 2007 and 2015, were selected for pathogenicity trials. Isolates were randomly selected to represent populations collected for microsatellite analyses (Additional file 1: Table S1). Pathogenicity tests were performed on six barley varieties (Barque, Franklin, Keel, Maritime, Skiff and Sloop) and four barley grass lines. Barley seed were obtained from the University of Adelaide’s Barley Breeding Program, National Variety Trials. Barley grass lines (Ald4, Cor5 & D’Ar5 from McLaren Vale, South Australia, and CSEPS7.1 from Canberra, Australian Capital Territory) were each established from a single seed [3]. Barley grass were identified based on morphological features by CC Linde. No permission was required for barley grass seed collection. Barley and barley grass lines were grown for 1 month in Hiko trays, one of each barley cultivar and barley grass lines per tray. The position of each seed line was randomly varied across trays. Barley and barley grass seeds were grown at 22 °C with 14 h days and 12 °C 10 h nights to reach the three leaf stage. Barley seeds were planted 5d after barley grass seeds to account for faster germination and growth of barley seed.

#### Inoculum preparation and inoculation

Each isolate was grown on Potato Dextrose Agar (PDA) at 22 °C for 10 days. The isolates were then transferred to V8 agar plates (15% clarified V8 juice, 1.5 g/L CaCO3, 15 g/L agar, pH 6.0–6.5) and incubated at 22 °C for 72 h in 12/12 h light/dark. Then, V8 plates were incubated at 18 °C for 72 h in the dark. Spores were harvested by vortexing multiple agar blocks in 25 mL 0.01% Tween20 in 50 mL tubes and filtered through eight layers of cheese-cloth. For isolates which did not produce spores with this method, spores were produced with a peanut hull extract agar medium [65]. Conidia were quantified using a haemocytometer and adjusted to a concentration of 10^4^ conidia/ml.

Each isolate was inoculated onto two trays of plants using ~ 100 mL spore suspension, spraying until runoff with a fine pressurised mister and incubated in a humidity chamber at 19 °C for 24 h in the dark. Plants were then incubated at 19 °C with a 12 h day length and night temperatures of 15 °C (±2 °C). Disease was scored after 2 weeks according to the Tekauz [66] rating scale ranging from 0 to 10, where 0 was most resistant and 10 highly susceptible. Scale values of 0–3 are considered resistant.

## Additional file

Additional file 1: **Table S1.** *Pyrenophora teres* f. *teres* populations studied from barley and barley grass in Australia. **Table S2.** *PhiPt* among *Pyrenophora teres* f.S*p. teres* populations. **Table S3.** Hierarchical Analyses of Molecular Variance partitioning of *Pyrenophora teres* f. *teres* SSR data among and within barley and barley grass hosts and populations. *P*-value estimates are based on 999 permutations. df = degrees of freedom, SS = sum of squares, MS = mean squared deviations. **Table S4.** Pathogenicity reaction of 20 *Pyrenophora teres* f. *teres* isolates from barley and barley grass on six barley varieties and four barley grass lines. (DOCX 48 kb)

## Data Availability

The dataset supporting the conclusions of this article is available in the Dryad repository https://datadryad.org/10.5061/dryad.q53v1p4.

## Abbreviations

$\overline{r}$_*d*_: Linkage disequilibrium index
AMOVA: Analysis of molecular variation
df: deegres of freedom
*E.5*: Evenness index
*eMLG*: The number of expected MLGs at the smallest sample size based on rarefaction
*H*: Shannon-Wiener index of MLG diversity
*H*_*e*_: Nei’s gene diversity
*I*: Shannon’s information index
*I*_*A*_: Index of association index
MLG: Multilocus genotype
MS: Mean squared deviations
N: Population size
*Na*: Number of alleles
PCoA: Principle coordinate analysis
PDA: Potato dextrose agar
*PhiPR*: Proportion of the total genetic variance that is due to the variance among populations within a region
*PhiPt*: Population differentiation
*PhiPT*: Proportion of the total genetic variance that is due to the variance among individuals within a variant
*PhiRT*: Proportion of the total genetic variance that is due to the variance between regions
Ptt: *Pyrenophora teres* f. *teres*
SE: Standard error
SS: Sum of squares
SSR: Simple sequence repeat
WA: Western Australia
