# Supplementary material for: Host specialisation and disparate evolution of Pyrenophora teres f. teres on barley and barley grass
Source: BMC Evol Biol. 2019 Jul 8;19:139. doi: 10.1186/s12862-019-1446-8 (PMC6615293; doi:10.1186/s12862-019-1446-8)
Supplement: Supplementary file 1 — Table S1. Pyrenophora teres f. teres populations studied from barley and barley grass in Australia. Table S2. PhiPt among Pyrenophora teres f.Sp. teres populations. Table S3. Hierarchical Analyses of Molecular Variance partitioning of Pyrenophora teres f. teres SSR data among and within barley and barley grass hosts and populations. P-value estimates are based on 999 permutations. df = degrees of freedom, SS = sum of squares, MS = mean squared deviations. Table S4. Pathogenicity reaction of 20 Pyrenophora teres f. teres isolates from barley and barley grass on six barley varieties and four barley grass lines. (DOCX 48 kb) [file 12862_2019_1446_MOESM1_ESM.docx]

**Supplementary material**

**Table S1**. *Pyrenophora teres* f. *teres* populations studied from barley and barley grass in Australia.

| **Population** | **Nearest town/area** | **State** | **Collected by** |
| --- | --- | --- | --- |
| **Barley grass** |  |  |  |
|  |  |  |  |
| BG_SA_PV | Port Vincent, York Peninsula | South Australia | CC Linde |
| BG_SA_DOW | Dowlinga | South Australia | CC Linde |
| BG_Barm | Barmedman | New South Wales | CC Linde |
| BG_Fin | Finley | New South Wales | CC Linde |
| BG_Nar | Narrandera | New South Wales | CC Linde |
| BG_Tem | Temora | New South Wales | CC Linde |
| BG_WW_GR | Wagga Wagga | New South Wales | CC Linde |
| BG_WW_MR | Wagga Wagga | New South Wales | CC Linde |
| BG_Kat | Katanning | Western Australia | CC Linde |
|  |  |  |  |
| **Barley** |  |  |  |
| Keel-45 | Yorke Peninsula | South Australia | H Wallwork |
| Keel-55 | Yorke Peninsula | South Australia | H Wallwork |
| Keel-63 | Yorke Peninsula | South Australia | H Wallwork |
| Keel-64 | Yorke Peninsula | South Australia | H Wallwork |
| Maritime-46 | Yorke Peninsula | South Australia | H Wallwork |
| Maritime-47 | Yorke Peninsula | South Australia | H Wallwork |
| Maritime-48 | Yorke Peninsula | South Australia | H Wallwork |
| Maritime-56 | Yorke Peninsula | South Australia | H Wallwork |
| Maritime-57 | Yorke Peninsula | South Australia | H Wallwork |
| Maritime-58 | Yorke Peninsula | South Australia | H Wallwork |
| Old_SA | - | South Australia | H Wallwork |
| SA_GP | - | South Australia | H Wallwork |
| SA_PV_B | Port Vincent, York Peninsula | South Australia | CC Linde |
| SA09 | - | South Australia | H Wallwork |
| NSW | - | New South Wales | CC Linde |
| Qld | - | Queensland | G Platz |
| Vic | - | Victoria | CC Linde |
| WA | - | Western Australia | S Gupta |

- Represent random collections from the specified state.

**Table S2.** *PhiPt* among *Pyrenophora teres* f.sp *teres* populations.

|  | Keel-45 | Keel-55 | Keel-63 | Keel-64 | Maritime-46 | Maritime-47 | Maritime-48 | Maritime-56 | Maritime-57 | Maritime-58 | OLD-SA | SA_GP | SA09 | NSW | Qld | Vic_GP | WA | SA_PV_B | BG_Barm | BG_Fin | BG_Kat | BG_Nar | BG_Tem | BG_WW_MR | BG_WW_GR | BG_SA_PV | BG_SA_DOW |
| --- | --- | --- | --- | --- | --- | --- | --- | --- | --- | --- | --- | --- | --- | --- | --- | --- | --- | --- | --- | --- | --- | --- | --- | --- | --- | --- | --- |
| Keel-45 | - | 0.034 | 0.001 | 0.001 | 0.001 | 0.001 | 0.001 | 0.001 | 0.001 | 0.001 | 0.001 | 0.006 | 0.001 | 0.001 | 0.001 | 0.001 | 0.001 | 0.001 | 0.001 | 0.001 | 0.001 | 0.001 | 0.001 | 0.001 | 0.001 | 0.001 | 0.001 |
| Keel-55 | 0.015 | - | 0.001 | 0.036 | 0.001 | 0.001 | 0.001 | 0.001 | 0.001 | 0.001 | 0.001 | 0.010 | 0.001 | 0.001 | 0.001 | 0.001 | 0.001 | 0.001 | 0.001 | 0.001 | 0.001 | 0.001 | 0.001 | 0.001 | 0.001 | 0.001 | 0.001 |
| Keel-63 | 0.073 | 0.070 | - | 0.001 | 0.001 | 0.001 | 0.001 | 0.001 | 0.001 | 0.001 | 0.001 | 0.003 | 0.001 | 0.001 | 0.001 | 0.009 | 0.001 | 0.001 | 0.001 | 0.001 | 0.001 | 0.001 | 0.001 | 0.001 | 0.001 | 0.001 | 0.001 |
| Keel-64 | 0.044 | 0.020 | 0.056 | - | 0.001 | 0.001 | 0.001 | 0.001 | 0.001 | 0.001 | 0.001 | 0.011 | 0.001 | 0.001 | 0.001 | 0.001 | 0.001 | 0.001 | 0.001 | 0.001 | 0.001 | 0.001 | 0.001 | 0.001 | 0.001 | 0.001 | 0.001 |
| Maritime-46 | 0.231 | 0.293 | 0.301 | 0.281 | - | 0.001 | 0.001 | 0.001 | 0.001 | 0.002 | 0.001 | 0.001 | 0.001 | 0.001 | 0.001 | 0.001 | 0.001 | 0.001 | 0.001 | 0.001 | 0.001 | 0.001 | 0.001 | 0.001 | 0.001 | 0.001 | 0.001 |
| Maritime-47 | 0.266 | 0.320 | 0.330 | 0.300 | 0.074 | - | 0.445 | 0.001 | 0.001 | 0.001 | 0.001 | 0.001 | 0.001 | 0.001 | 0.001 | 0.001 | 0.001 | 0.001 | 0.001 | 0.001 | 0.001 | 0.001 | 0.001 | 0.001 | 0.001 | 0.001 | 0.001 |
| Maritime-48 | 0.264 | 0.322 | 0.339 | 0.306 | 0.050 | 0.000 | - | 0.001 | 0.001 | 0.001 | 0.001 | 0.001 | 0.001 | 0.001 | 0.001 | 0.001 | 0.001 | 0.001 | 0.001 | 0.001 | 0.001 | 0.001 | 0.001 | 0.001 | 0.001 | 0.001 | 0.001 |
| Maritime-56 | 0.233 | 0.272 | 0.277 | 0.262 | 0.086 | 0.126 | 0.120 | - | 0.001 | 0.001 | 0.001 | 0.001 | 0.001 | 0.001 | 0.001 | 0.001 | 0.001 | 0.001 | 0.001 | 0.001 | 0.001 | 0.001 | 0.001 | 0.001 | 0.001 | 0.001 | 0.001 |
| Maritime-57 | 0.228 | 0.291 | 0.290 | 0.265 | 0.035 | 0.059 | 0.044 | 0.108 | - | 0.103 | 0.001 | 0.001 | 0.001 | 0.001 | 0.001 | 0.001 | 0.001 | 0.001 | 0.001 | 0.001 | 0.001 | 0.001 | 0.001 | 0.001 | 0.001 | 0.001 | 0.001 |
| Maritime-58 | 0.212 | 0.273 | 0.292 | 0.253 | 0.031 | 0.062 | 0.049 | 0.094 | 0.009 | - | 0.001 | 0.001 | 0.002 | 0.001 | 0.001 | 0.001 | 0.001 | 0.001 | 0.001 | 0.001 | 0.001 | 0.001 | 0.001 | 0.001 | 0.001 | 0.001 | 0.001 |
| OLD-SA | 0.169 | 0.172 | 0.133 | 0.171 | 0.327 | 0.366 | 0.370 | 0.305 | 0.314 | 0.295 | - | 0.001 | 0.001 | 0.017 | 0.001 | 0.008 | 0.001 | 0.001 | 0.001 | 0.001 | 0.001 | 0.001 | 0.001 | 0.001 | 0.001 | 0.001 | 0.001 |
| SA_GP | 0.039 | 0.044 | 0.065 | 0.046 | 0.248 | 0.270 | 0.279 | 0.239 | 0.230 | 0.218 | 0.127 | - | 0.069 | 0.001 | 0.001 | 0.002 | 0.029 | 0.002 | 0.001 | 0.001 | 0.001 | 0.001 | 0.001 | 0.001 | 0.001 | 0.001 | 0.001 |
| SA09 | 0.086 | 0.129 | 0.128 | 0.114 | 0.108 | 0.117 | 0.132 | 0.117 | 0.088 | 0.087 | 0.137 | 0.043 | - | 0.001 | 0.001 | 0.002 | 0.001 | 0.001 | 0.001 | 0.001 | 0.001 | 0.001 | 0.001 | 0.001 | 0.001 | 0.001 | 0.001 |
| NSW | 0.192 | 0.188 | 0.165 | 0.200 | 0.346 | 0.381 | 0.384 | 0.340 | 0.336 | 0.312 | 0.038 | 0.140 | 0.146 | - | 0.009 | 0.001 | 0.001 | 0.001 | 0.001 | 0.001 | 0.001 | 0.001 | 0.001 | 0.001 | 0.001 | 0.001 | 0.001 |
| Qld | 0.181 | 0.185 | 0.173 | 0.184 | 0.292 | 0.323 | 0.323 | 0.280 | 0.279 | 0.255 | 0.060 | 0.120 | 0.110 | 0.032 | - | 0.001 | 0.001 | 0.001 | 0.001 | 0.001 | 0.001 | 0.001 | 0.001 | 0.001 | 0.001 | 0.001 | 0.001 |
| Vic_GP | 0.091 | 0.129 | 0.058 | 0.138 | 0.323 | 0.368 | 0.375 | 0.306 | 0.310 | 0.295 | 0.066 | 0.105 | 0.123 | 0.091 | 0.134 | - | 0.001 | 0.001 | 0.001 | 0.001 | 0.001 | 0.001 | 0.001 | 0.001 | 0.001 | 0.001 | 0.001 |
| WA | 0.039 | 0.047 | 0.068 | 0.045 | 0.213 | 0.250 | 0.252 | 0.212 | 0.218 | 0.204 | 0.174 | 0.021 | 0.094 | 0.189 | 0.175 | 0.115 | - | 0.001 | 0.001 | 0.001 | 0.001 | 0.001 | 0.001 | 0.001 | 0.001 | 0.001 | 0.001 |
| SA_PV_B | 0.071 | 0.084 | 0.104 | 0.092 | 0.246 | 0.273 | 0.277 | 0.249 | 0.238 | 0.225 | 0.134 | 0.049 | 0.079 | 0.137 | 0.136 | 0.096 | 0.081 | - | 0.001 | 0.001 | 0.001 | 0.001 | 0.001 | 0.001 | 0.001 | 0.001 | 0.001 |
| BG_Barm | 0.415 | 0.429 | 0.447 | 0.432 | 0.524 | 0.521 | 0.529 | 0.492 | 0.502 | 0.488 | 0.460 | 0.444 | 0.401 | 0.437 | 0.402 | 0.472 | 0.426 | 0.385 | - | 0.106 | 0.001 | 0.061 | 0.286 | 0.001 | 0.001 | 0.001 | 0.001 |
| BG_Fin | 0.425 | 0.439 | 0.464 | 0.442 | 0.540 | 0.540 | 0.549 | 0.506 | 0.518 | 0.500 | 0.467 | 0.458 | 0.421 | 0.448 | 0.415 | 0.494 | 0.433 | 0.394 | 0.014 | - | 0.001 | 0.015 | 0.100 | 0.001 | 0.001 | 0.001 | 0.001 |
| BG_Kat | 0.499 | 0.513 | 0.538 | 0.534 | 0.623 | 0.629 | 0.632 | 0.596 | 0.606 | 0.594 | 0.548 | 0.561 | 0.532 | 0.533 | 0.501 | 0.586 | 0.513 | 0.475 | 0.398 | 0.388 | - | 0.001 | 0.001 | 0.001 | 0.001 | 0.001 | 0.001 |
| BG_Nar | 0.431 | 0.442 | 0.459 | 0.439 | 0.530 | 0.525 | 0.533 | 0.501 | 0.508 | 0.496 | 0.469 | 0.450 | 0.420 | 0.446 | 0.419 | 0.483 | 0.438 | 0.406 | 0.015 | 0.026 | 0.378 | - | 0.165 | 0.001 | 0.001 | 0.001 | 0.001 |
| BG_Tem | 0.396 | 0.411 | 0.433 | 0.413 | 0.526 | 0.524 | 0.534 | 0.489 | 0.500 | 0.483 | 0.444 | 0.404 | 0.370 | 0.411 | 0.376 | 0.464 | 0.409 | 0.354 | 0.013 | 0.030 | 0.464 | 0.019 | - | 0.001 | 0.001 | 0.001 | 0.001 |
| BG_WW_MR | 0.420 | 0.432 | 0.461 | 0.435 | 0.536 | 0.536 | 0.544 | 0.504 | 0.515 | 0.498 | 0.459 | 0.445 | 0.416 | 0.438 | 0.407 | 0.488 | 0.430 | 0.389 | 0.156 | 0.140 | 0.458 | 0.148 | 0.161 | - | 0.001 | 0.001 | 0.001 |
| BG_WW_GR | 0.388 | 0.402 | 0.423 | 0.403 | 0.502 | 0.497 | 0.508 | 0.470 | 0.482 | 0.467 | 0.434 | 0.401 | 0.374 | 0.408 | 0.383 | 0.428 | 0.397 | 0.355 | 0.114 | 0.122 | 0.408 | 0.128 | 0.109 | 0.072 | - | 0.001 | 0.001 |
| BG_SA_PV | 0.326 | 0.344 | 0.355 | 0.341 | 0.453 | 0.452 | 0.464 | 0.417 | 0.430 | 0.413 | 0.355 | 0.303 | 0.278 | 0.315 | 0.299 | 0.337 | 0.334 | 0.264 | 0.180 | 0.184 | 0.332 | 0.192 | 0.161 | 0.181 | 0.085 | - | 0.001 |
| BG_SA_DOW | 0.395 | 0.407 | 0.428 | 0.415 | 0.505 | 0.498 | 0.508 | 0.474 | 0.486 | 0.474 | 0.433 | 0.408 | 0.376 | 0.409 | 0.388 | 0.426 | 0.412 | 0.357 | 0.187 | 0.206 | 0.406 | 0.204 | 0.191 | 0.204 | 0.097 | 0.128 | - |

**Table S3.** Hierarchical Analyses of Molecular Variance partitioning of *Pyrenophora teres* f. *teres* SSR data among and within barley and barley grass hosts and populations. *P*-value estimates are based on 999 permutations. df = degrees of freedom, SS = sum of squares, MS = mean squared deviations.

**Barley**

| **Source** | **df** | **SS** | **MS** | **Estimated variance** | **Percentage variance** | **AMOVA statistics** | ***P*** |
| --- | --- | --- | --- | --- | --- | --- | --- |
| Among populations | 17 | 677.297 | 39.841 | 1.123 | 19% | *PhiPT* = 0.194 | 0.001 |
| Within populations | 549 | 2568.852 | 4.679 | 4.679 | 81% |  |  |

**Barley grass**

| **Source** | **df** | **SS** | **MS** | **Estimated variance** | **Percentage variance** | **AMOVA statistics** | ***P*** |
| --- | --- | --- | --- | --- | --- | --- | --- |
| Among populations | 10 | 275.760 | 27.576 | 0.919 | 22% | *PhiPT* = 0.218 | 0.001 |
| Within populations | 287 | 948.398 | 3.305 | 3.305 | 78% |  |  |

**Table S4.** Pathogenicity reaction of 20 *Pyrenophora teres* f.sp *teres* isolates from barley and barley grass on six barley varieties and four barley grass lines.

| Isolate | Population | Barley variety | | | | | |  | | Barley grass line | | | | |
| --- | --- | --- | --- | --- | --- | --- | --- | --- | --- | --- | --- | --- | --- | --- |
|  |  | **Barque** | **Franklin** | **Keel** | **Maritime** | **Skiff** | **Sloop** | |  | | **Ald4** | **Cor5** | **CSEPS7.1** | **D'Ar5** |
| 30 | Keel-45 | 3 | 6 | 7 | 9 | 7 | 6 | |  | | 1 | 1 | 0 | 0 |
| 382 | Keel-64 | 3 | 4 | 6 | 4 | 8 | 7 | |  | | 1 | 1 | 0 | 1 |
| 67 | Maritime-46 | 4 | 3 | 2 | 8 | 8 | 7 | |  | | 0 | 1 | 1 | 0 |
| 319 | Maritime-58 | 5 | 2 | 3 | 7 | 8 | 5 | |  | | 1 | 1 | 1 | 1 |
| 646 | NSW | 5 | 2 | 3 | 4 | 5 | 2 | |  | | 1 | 1 | 0 | 1 |
| 647 | NSW | 6 | 5 | 6 | 3 | 3 | 6 | |  | | 1 | 1 | 1 | 1 |
| 542 | WA | 3 | 4 | 5 | 4 | 5 | 5 | |  | | 0 | 1 | 1 | 1 |
| 547 | WA | 4 | 5 | 6 | 4 | 4 | 4 | |  | | 1 | 0 | 0 | 1 |
| 1335 | SA_PV_B | 6 | 6 | 3 | 7 | 8 | 6 | |  | | 1 | 1 | 1 | 0 |
| 1341 | SA_PV_B | 5 | 6 | 6 | 5 | 4 | 8 | |  | | 0 | 0 | 1 | 1 |
|  |  |  |  |  |  |  |  | |  | |  |  |  |  |
| 962 | BG_Barm | 1 | 1 | 1 | 1 | 0 | 1 | |  | | 2 | 5 | 4 | 4 |
| 1543 | BG_Dow | 1 | 0 | 1 | 1 | 1 | 0 | |  | | 3 | 2 | 3 | 5 |
| 900 | BG_Fin | 1 | 1 | 1 | 1 | 2 | 1 | |  | | 5 | 2 | 4 | 6 |
| 1577 | BG_Kat | 0 | 1 | 1 | 1 | 1 | 1 | |  | | 7 | 5 | 2 | 3 |
| 741 | BG_Nar | 1 | 1 | 0 | 0 | 1 | 1 | |  | | 6 | 4 | 5 | 4 |
| 1409 | BG_SA_PV | 0 | 0 | 1 | 1 | 1 | 0 | |  | | 3 | 5 | 4 | 7 |
| 693 | BG_Tem | 1 | 1 | 1 | 1 | 1 | 1 | |  | | 5 | 6 | 4 | 2 |
| 694 | BG_Tem | 1 | 0 | 1 | 1 | 1 | 1 | |  | | 4 | 7 | 5 | 4 |
| 1195 | BG_WW_GR | 1 | 1 | 1 | 0 | 1 | 1 | |  | | 3 | 3 | 5 | 6 |
| 1143 | BG_WW_MR | 1 | 1 | 1 | 1 | 2 | 1 | |  | | 5 | 2 | 5 | 3 |
